# PSF Knockdown Enhances Apoptosis via Downregulation of LC3B in Human Colon Cancer Cells

**DOI:** 10.1155/2013/204973

**Published:** 2013-10-31

**Authors:** Tamotsu Tsukahara, Yoshikazu Matsuda, Hisao Haniu

**Affiliations:** ^1^Department of Hematology and Immunology, Kanazawa Medical University, 1-1 Daigaku, Uchinada, Ishikawa 920-0293, Japan; ^2^Clinical Pharmacology Educational Center, Nihon Pharmaceutical University, Ina-machi, Saitama 362-0806, Japan; ^3^Department of Orthopaedic Surgery, Shinshu University School of Medicine, 3-1-1 Asahi, Matsumoto, Nagano 390-8621, Japan

## Abstract

Our previous study demonstrated that PTB-associated splicing factor (PSF) is an important regulator of cell death and plays critical roles in the survival and growth of colon cancer cells. However, the molecular mechanism that activates these downstream signaling events remains unknown. To address this issue, we investigated the effects of PSF knockdown in two different colon cancer cell lines, DLD-1 and HT-29. We found that knockdown of PSF markedly decreased the autophagic molecule LC3B in DLD-1 cells but not in HT-29 cells. Furthermore, DLD-1 cells were more susceptible to PSF knockdown-induced cell growth inhibition and apoptosis than HT-29 cells. This susceptibility is probably a result of LC3B inhibition, given the known relationship between autophagy and apoptosis. C3B is associated with a number of physiological processes, including cell growth and apoptotic cell death. Our results suggest that autophagy is inhibited by PSF knockdown and that apoptosis and cell growth inhibition may act together to mediate the PSF-LC3B signaling pathway. Furthermore, we found that the peroxisome proliferator-activated receptor gamma (PPAR**γ**)-PSF complex induced LC3B downregulation in DLD-1 cells. The results of this study identify a new physiological role for the PSF-LC3B axis as a potential endogenous modulator of colon cancer treatment.

## 1. Introduction

Most cancer cells are resistant to chemotherapy because of genetic mutations [1, 2] or deletions in apoptotic molecules [3, 4]. Therefore, to improve the therapeutic efficacy of cancer treatment, it is important to find alternative approaches to induce cell death. To this end, we searched for proteins that interact with peroxisome proliferator-activated receptor gamma (PPAR*γ*), which is expressed at significant levels in human colon cancer cells and tissues [5]. Activation of PPAR*γ* is associated with cell cycle progression and the expression of genes that promote cell differentiation. We identified PTB-associated splicing factor (PSF) as a novel PPAR*γ*-interacting protein and demonstrated that PSF is involved in several important regulatory steps of colon cancer cell proliferation [6]. PSF is a multifunctional protein involved in transcription regulation, pre-mRNA processing, and DNA repair [7]. A recent study showed that PSF belongs to a family of putative tumor-suppressor proteins that contain an RNA-binding domain and a DNA-binding domain [8, 9]. Wang et al. reported that PSF has a central role in the reversible regulation of cell proliferation and tumorigenesis [10]. Alteration in the expression of PSF and its binding partners may have potential as a therapeutic strategy against cancer [11]. 

In our previous study, we showed that PSF is involved in several important regulatory steps of colon cancer cell proliferation and demonstrated that PSF knockdown induced apoptosis in human colon cancer cells [6]. Expression of PSF siRNA significantly suppressed proliferation and induced apoptosis via activation of caspase-3 in colon cancer cells. Furthermore, in DLD-1 cells, voltage-dependent anion selective channel protein 2 (VDAC2) was upregulated under PSF knockdown conditions. These results suggest that PSF is an important regulator of cell death and plays critical roles in the survival and growth of colon cancer cells. However, how the various activities of PSF are regulated in colon cancer cells is not yet clear. In our previous study, DLD-1 cells appeared as empty, lucent spaces in phase contrast images after PSF siRNA transfection. This cell vacuolation suggests that PSF knockdown affected autophagy in DLD-1 colon cancer cells. 

Autophagy, first described in 1963 [12], is a tightly regulated cellular process of bulk cytoplasmic and organelle degradation [13–15]. Common to nearly all eukaryotes, autophagy serves as a lysosomal degradation pathway for recycling intracellular components such as protein aggregates and damaged or dysfunctional intracellular organelles [16, 17]. Nutrient deprivation is among the best-characterized inducers of autophagy [18]. Decreased metabolism leads to the induction of autophagy to generate nutrients from intracellular components. In dividing cancer cells, metabolic stress as a result of insufficient nutrient and oxygen supply induces autophagy as an alternative source of metabolites [19]. Inhibition of autophagy by an autophagic-specific inhibitor or an RNAi method can trigger apoptosis, and suppression of autophagy by knockdown of the autophagic protein LC3B promotes apoptosis and caspase-3 activation [20]. These observations suggest that the level of LC3B expression might correlate with the proliferation potential of colon cancer cells. Whereas increased autophagy is expected to promote tumor growth, reduced autophagy might provide a useful way to limit tumor growth [21]. However, the physiologic relevance of autophagy in tumor formation and progression is still controversial. Based on previous observations, we hypothesized that PSF regulates cell death and proliferation in colon cancer cells by influencing autophagy. Autophagy and apoptosis are both highly regulated biological processes with essential roles in homeostasis and disease. Autophagy has been described as a mechanism of cell death, although the precise mechanisms that link autophagy and cell death are not fully understood. We investigated autophagy in colon cancer cells by assessing the expression of the autophagic marker LC3B and its effects on cell death and proliferation. This study is the first to describe the effects of PSF on cell proliferation, tumor growth, and apoptosis associated with LC3B.

## 2. Materials and Methods

### 2.1. Reagents

Mouse monoclonal anti-PSF antibody (sc-271796), rabbit polyclonal anti-PPAR*γ* antibody (sc-7196), mouse monoclonal anti-*β*-actin antibody (sc-32059), PSF siRNA (sc-38304), LC3B siRNA (sc-43390), and control siRNA (sc-37007) were purchased from Santa Cruz Biotechnology (Santa Cruz, CA, USA). Rabbit polyclonal anti-LC3B antibody (no. 2775) was purchased from Cell Signaling Technology (Danvers, MA, USA). Full-length human LC3B cDNA was purchased from IMAGE Clone Consortium (IMAGE number: 3623259). A PCR product amplified using Tks Gflex DNA polymerase (Takara, Shiga, Japan) was inserted into a pcDNA3.1(+) vector (Invitrogen, Carlsbad, CA, USA). 

### 2.2. Cell Culture

Human colorectal cancer cell lines, DLD-1 and HT-29, were obtained from the American Type Culture Collection (Manassas, VA, USA). Cells were grown in Dulbecco's modified Eagle's medium (DMEM; Nacalai Tesuque, Kyoto, Japan) containing 10% (v/v) fetal bovine serum (FBS) at 37°C in a humidified incubator with 5% CO_2_. 3-Methyladenine (3-MA) was purchased from Santa Cruz Biotechnology. 

### 2.3. Western Blotting

Cells were washed 2 times with ice-cold PBS and solubilized using an EzRIPA Lysis kit (ATTO, Tokyo, Japan). The cell lysate was centrifuged at 14,000 ×g for 5 min, and the protein in the supernatant was quantified using a Protein Quantification Kit-Rapid (ATTO). Total protein was diluted 1 : 4 with Lane Marker Reducing Sample Buffer (ThermoFisher Scientific, Waltham, MA, USA) and boiled for 5 min. Protein was then separated on 10% SDS-PAGE and transferred to a PVDF membrane (GE Healthcare, Piscataway, NJ, USA). The membrane was blocked with 5% skim milk in Tris-buffered saline (TBS) with 0.1% Tween 20 (pH 7.6) for 1 h at room temperature and probed with primary rabbit anti-LC3B antibody at 4°C overnight. After the membrane was washed, it was incubated with secondary anti-rabbit antibody (GE Healthcare, Little Chalfont, UK) for 1 h at room temperature and then developed with the EzWestLumi plus chemiluminescent detection reagent (ATTO). 

### 2.4. Measurement of Cell Proliferation

PSF was knocked down in DLD-1 cells, which were seeded in 96-well culture plates (1 × 10^4^ cells/well). Cell proliferation was determined using Cell Counting Kit-8 (Dojindo, Kumamoto, Japan). Ten microliters of Cell Counting Kit-8 solution were added to the medium and incubated for 2 h in an incubator with a 5% CO_2_ atmosphere. The amount of orange formazan dye produced was calculated by measuring the absorbance at 450 nm in a microplate reader (Awareness Technology, Inc., Palm City, FL, USA). 

### 2.5. Quantitative Real-Time PCR Analysis

Total RNA was prepared from HT-29 and DLD-1 cells using NucleoSpin RNA II (Takara). cDNA was then synthesized using 0.5 *μ*g of total RNA and the ReverTra Ace qPCR RT Kit (Toyobo, Osaka, Japan), as recommended by the manufacturer. mRNA levels were quantified using an ECO Real-Time PCR system (Illumina, Inc., San Diego, CA, USA) and SYBR Green Real-time PCR Master Mix-Plus (Toyobo) with the following primer pair sets: LC3B, 5′-GAGAAGCAGCTTCCTGTTCTGG-3′ (F) and 5′-GTGTCCGTTCACCAACAGGAAG-3′ (R); 18S rRNA, 5′-CAGCCACCCGAGATTGAGCA-3′ (F); and 5′-TAGTAGCGACGGGCGGTGTG-3′ (R). All reactions were performed in a 10 *μ*L volume using 48-well PCR plates (Illumina). The cycling conditions were 95°C for 10 min (polymerase activation), followed by 40 cycles of 95°C for 15 sec, 55°C for 15 sec, and 72°C for 30 sec. In order to determine which housekeeping genes were most suitable for the subsequent normalization of data, we initially selected 3 candidates, GAPDH, *β*-actin, and 18S-rRNA, which are commonly used for internal controls in mammalian cells. After amplification, the samples were slowly heated from 55°C to 95°C with continuous reading of fluorescence to obtain a melting curve. The relative mRNA level was calculated by using the arithmetic formula 2^−ΔΔCq^, where ΔCq is the difference between the threshold cycle of a given target cDNA and an endogenous reference cDNA. Derivations of the formulas and validation tests have been described in Applied Biosystems User Bulletin no. 2. 

### 2.6. Small Interfering RNA

PSF expression was inhibited in HT-29 and DLD-1 cells by transfection with small interfering RNA (siRNA) targeting PSF (Santa Cruz Biotechnology), using Lipofectamine RNAiMAX (Invitrogen). Cells were plated onto 6-well plates (Iwaki, Tokyo, Japan) at a density of 5 × 10^4^ cells per well in DMEM containing 10% FBS. Cells were transfected with 100 pmol/mL of mRNA-specific siRNA or scrambled control siRNA. The reduction in PSF or LC3B levels was confirmed by western blot analysis. 

### 2.7. Statistical Analysis

Student's *t*-test was used for statistical comparisons. Differences were considered significant when the *P* value was below 0.05. 

## 3. Results and Discussion

In the present study, we showed that LC3B is downregulated by PSF knockdown. Decreased expression of LC3B in colon cancer cells induced apoptosis. This finding suggests that PSF-mediated LC3B downregulation plays a novel role in the regulation of cell proliferation and apoptosis, which presents a potential therapeutic strategy for colon cancer. We have previously shown that DLD-1 cells are more susceptible to PSF knockdown-induced cell death than HT-29 cells [6]. Moreover, PSF knockdown also induced morphological changes associated with apoptosis, that is, cell shrinkage and condensation of nuclear chromatin, in DLD-1 cells, but not HT-29 cells. Furthermore, PSF knockdown induced vacuolation in DLD-1 cells but not in HT-29 cells. To investigate autophagy in the two cell lines, we used LC3B as a marker of autophagy. 

**Figure 1:**
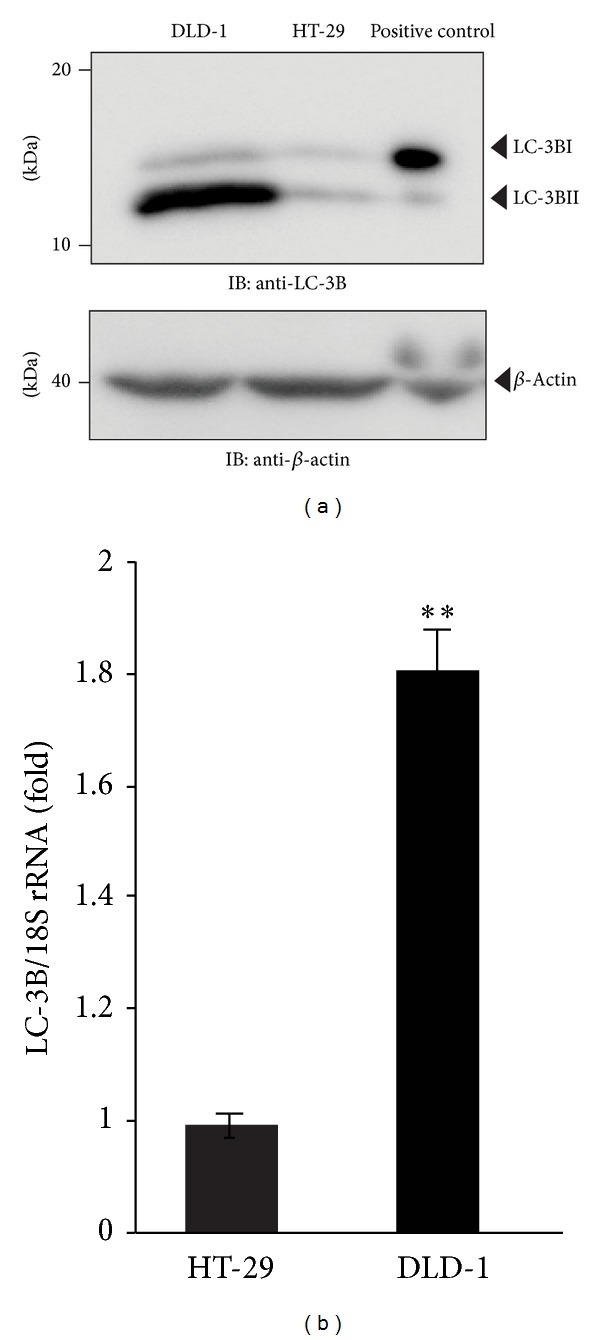
Comparison of endogenous LC3B protein expression. (a) Representative western blot of LC3-I and LC3-II expression. Whole cell lysate (50 *μ*g) was analyzed by SDS-PAGE and visualized with enhanced chemiluminescence as described in the experimental procedures. (b) Real-time PCR measurement of LC3B mRNA expression in DLD-1 and HT-29 cells. The relative LC3B levels normalized to 18S rRNA are expressed as the mean ± SEM (*n* = 3), **P < 0.01.

During autophagy, LC3B-I is converted to LC3B-II through lipidation by Atg7 and Atg3, which allows LC3 to associate with autophagic vesicles [22]. Abnormal expression of LC3B has been reported in human colon cancer [23]. LC3B has been used as a marker of autophagy in recent studies [24–26]. When autophagy is not activated, LC3B is localized in the cytoplasm. However, upon initiation of autophagy under amino acid deprivation [27], LC3B associates with the isolation membrane. Cleavage of LC3B at the carboxyl terminus immediately following synthesis yields the cytosolic LC3B-I form. During autophagy, LC3B-I is converted to LC3B-II through lipidation by Atg7 and Atg3, which allows LC3B to associate with autophagic vesicles [22]. After autophagosomes are formed, they undergo a stepwise maturation process in which they engulf organelles, fuse with lysosomes, and mature into autolysosomes with lysosomal enzymes [16]. We first examined the expression of LC3B mRNA and protein in DLD-1 and HT-29 cells. First, we examined the expression level of LC3B in two different colon cancer cell lines, DLD-1 and HT-29. Interestingly, as shown in Figure 1(a), LC3B protein was expressed at higher levels in DLD-1 cells than in HT-29 cells. The expression of LC3B-II protein was consistent with that of LC3B mRNA (Figure 1(b)): expression of LC3B-II protein was significantly higher in DLD-1 cells than in HT-29 cells. These results suggest that DLD-1 cells express a high level of LC3B-II protein under basal conditions.

**Figure 2:**
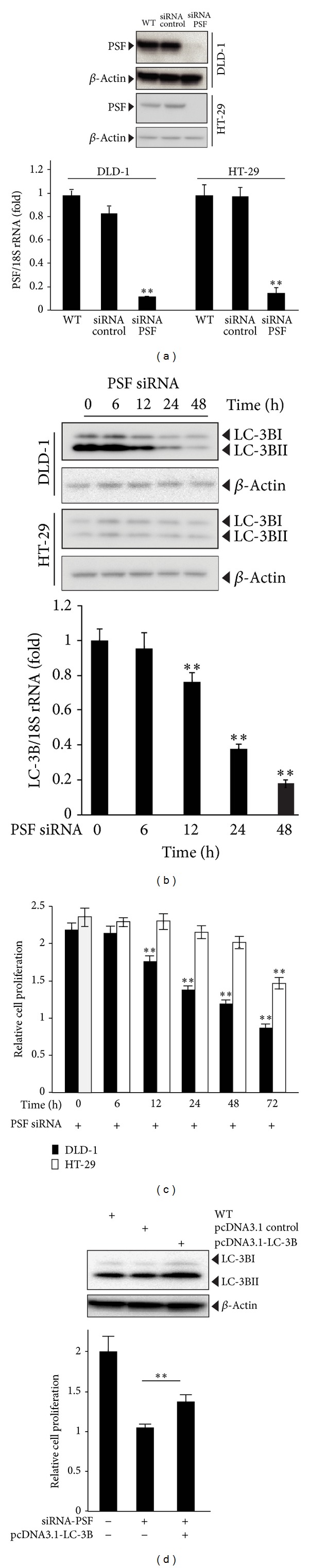
PSF knockdown decreases LC3B expression and inhibits the proliferation of DLD-1 cells. (a) Expression of PSF was knocked down in DLD-1 and HT-29 cells. Top panel: total protein was extracted from untransfected (WT), control siRNA-transfected, or PSF siRNA-transfected cells. Forty-eight hours later, whole-cell lysates were subjected to western blot analysis for PSF. Incubation with an anti-*β*-actin antibody was used as a protein-loading control. Bottom panel: Real-Time PCR measurement of PSF mRNA expression in DLD-1 and HT-29 cells. The relative PSF levels normalized to 18S rRNA are expressed as the mean ± SEM (*n* = 3), **P < 0.01. (b) Decreased LC3B expression after PSF knockdown in DLD-1 and HT-29 cells. Top panel: protein was analyzed by SDS-PAGE at the indicated times after PSF siRNA transfection, subjected to western blotting, and visualized with an enhanced chemiluminescence reagent. Each lane was loaded with 20 *μ*g of whole-cell lysate. *β*-actin was used as an internal loading control and was detected using mouse anti-*β*-actin antibody. Bottom panel: (c) time-dependent cell growth inhibition was measured using Cell Counting Kit-8 at 6, 12, 24, 48, and 72 h after siRNA transfection. An equal number of cells (1 × 10^5^ cells/well) were seeded in 6-well plates and then incubated overnight at 37°C in an incubator with 5% CO_2_. Cell Counting Kit-8 was added to the medium and incubated for 2 h in the incubator. The amount of orange formazan dye generated was calculated by measuring the absorbance at 450 nm in a microplate reader. Data are expressed as mean ± SEM (*n* = 3; **P < 0.01). (d) Top panel: protein was extracted from untransfected (WT), control pcDNA3.1-transfected, or pcDNA3.1-LC3B-transfected cells. Bottom panel: PSF knocked down DLD-1 cells were transfected with LC3B plasmid and incubated for 24 h. Cell proliferation was measured using Cell Counting Kit-8.

There are two major classes of programmed cell death, apoptotic cell death (type 1) and autophagic cell death (type 2), both of which are defined by morphological criteria [21]. Autophagic cell death is morphologically characterized by an accumulation of autophagic vacuoles. Inhibition of autophagy by an autophagic-specific inhibitor can trigger apoptosis. To verify the type of cell death induced by PSF knockdown, we first examined whether autophagy directly contributes to the survival of DLD-1 cells and HT-29 cells under nutrient-rich conditions. To evaluate the effects of PSF expression and autophagy regulation, PSF expression was knocked down using siRNA. As shown in Figure 2(a), real-time quantitative RT-PCR analysis and western blot showed that PSF mRNA and protein, respectively, were knocked down in DLD-1 cells transfected with PSF siRNA. Western blot analysis also showed that transfection with PSF siRNA decreased the expression of LC3B in DLD-1 cells, but not in HT-29 cells, in a time-dependent manner (Figure 2(b)). These results suggest that LC3B is downregulated by PSF knockdown. We also determined the effect of PSF knockdown on cell proliferation. As shown in Figures 2(c) and 2(d), cell proliferation was decreased by PSF knockdown, and this inhibitory effect was reversed by LC3B overexpression. Thus, we observed distinct cell-type-specific differences associated with the LC3B-PSF interaction and demonstrated a direct functional effect of PSF on cell proliferation and apoptosis.

**Figure 3:**
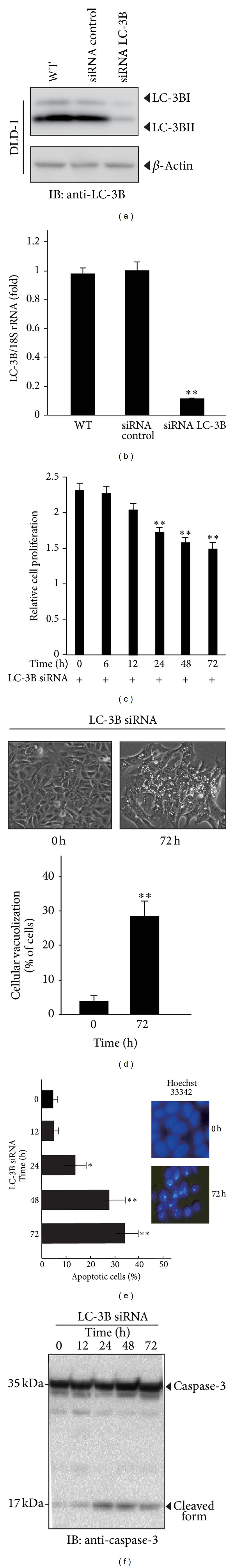
LC3B knockdown induces apoptosis in DLD-1 cells. (a) Expression of LC3B was knocked down in DLD-1 cells. Total protein was extracted from untransfected (WT), control siRNA-transfected, or LC3B siRNA-transfected cells. Forty-eight hours later, whole-cell lysates were subjected to western blot analysis for LC3B. Incubation with an anti-*β*-actin antibody was used as a protein-loading control. (b) Real-time PCR measurement of LC3B mRNA expression in DLD-1 cells. The relative LC3B levels normalized to 18S rRNA are expressed as the mean ± SEM (*n* = 3), **P < 0.01. (c) Time-dependent cell growth inhibition was measured using Cell Counting Kit-8 at 6, 12, 24, 48, and 72 h after LC3B siRNA transfection. Data are expressed as mean ± SEM (*n* = 3; **P < 0.01). (d) Vacuolated cells were analyzed and counted as described in the experimental procedures. At least 3 fields of cells per sample were counted and tabulated. Data are expressed as mean ± SEM (*n* = 3; **P < 0.01). (e) siRNA transfected DLD-1 cells were stained with Hoechst 33342 and analyzed by fluorescence microscopy. Apoptotic nuclei stained more brightly than nuclei in untransfected cells or siRNA control-transfected cells. At least 5 fields of cells per sample were counted and tabulated. Values are expressed as the mean ± SEM (*n* = 5), **P < 0.05 based on Student's *t*-test. (f) LC3B siRNA transfected DLD-1 cells were collected in RIPA buffer, and 50 *μ*g of protein was loaded for SDS-PAGE. Protein was analyzed by western blot using an anti-caspase-3 antibody to assess apoptosis.

**Figure 4:**
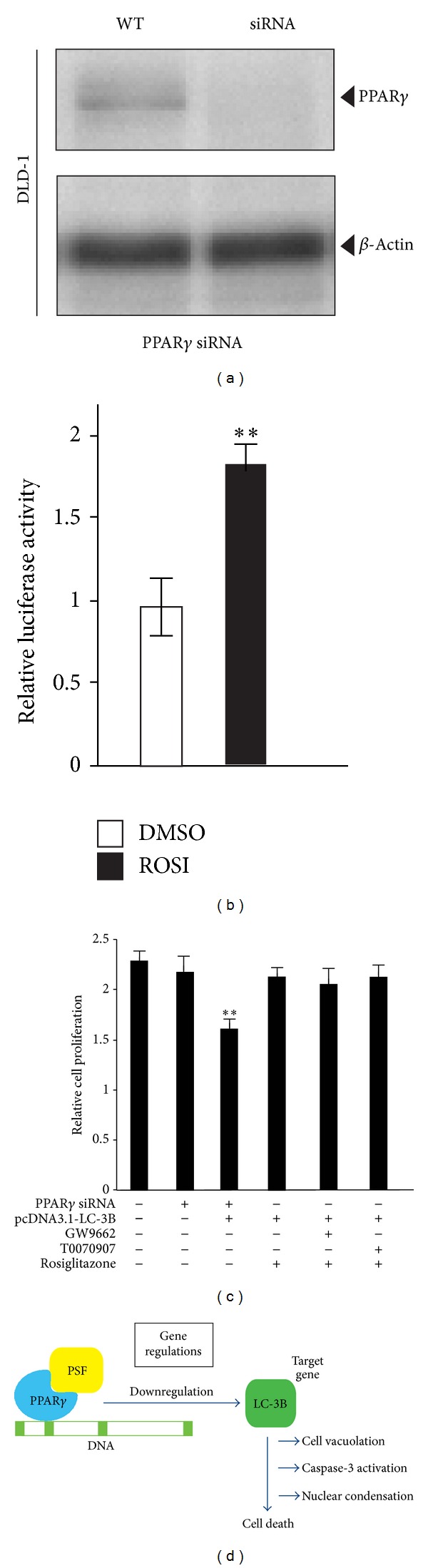
PPAR*γ* activation is not involved in DLD-1 cell proliferation. (a) PPAR*γ* was knocked down in DLD-1 cells. Total protein was extracted from untransfected (WT) and PPAR*γ* siRNA-transfected cells. Forty-eight hours later, whole-cell lysates were subjected to western blot analysis for PPAR*γ*. Incubation with an anti-*β*-actin antibody was used as a protein-loading control. (b) Effect of rosiglitazone on reporter activation in DLD-1 cells. Cells were transiently transfected with a pGL3-PPRE-acyl-CoA oxidase luciferase reporter vector. The cells were treated with 10 *μ*M rosiglitazone (ROSI) for 20 h. Luciferase activity was normalized to luciferase activity. Data are expressed as mean ± SEM (*n* = 4; **P < 0.01). (c) PPAR*γ* activation is not involved in PSF-LC3B downregulation in DLD-1 proliferation. Cell growth inhibition was measured using Cell Counting Kit-8 at 72 h after treatment with the vehicle, 10 *μ*M rosiglitazone, 10 *μ*M T0070907, or 10 *μ*M GW9662. Data are expressed as mean ± SEM (*n* = 3; **P < 0.01). (d) Schematic representation of the proposed mechanism of PSF-LC3B action in DLD-1 cells.

Suppression of autophagy by LC3B knockdown has been shown to promote apoptosis and caspase-3 activation [20]. Therefore, we investigated the effect of LC3B expression on apoptosis. To evaluate the effects of LC3B on the proliferation of DLD-1 cells, the expression of LC3B was knocked down using siRNA. Knockdown of LC3B expression in DLD-1 cells using siRNA was effective, as evidenced by western blot analysis using an anti-LC3B antibody (Figure 3(a)). Real-time quantitative RT-PCR analysis showed that LC3B siRNA reduced LC3B mRNA levels by 80%–90%, compared to the levels in untransfected (WT) control cells (Figure 3(b)). We then determined the effect of LC3B knockdown on cell proliferation. As shown in Figure 4(c), LC3B knockdown decreased cell proliferation. Moreover, DLD-1 cells appeared as an empty lucent space in phase contrast images at 72 h after siRNA LC3B transfection (Figure 3(d)). At 72 h after transfection, approximately 30% of the cells showed extensive vacuolization of the cytoplasm. Vacuolation of cells increased in number and size, occupying increasingly larger areas of the cytoplasm in a time-dependent manner. The decreased cell proliferation observed in conjunction with the morphological observations suggests that DLD-1 cells treated with LC3B siRNA undergo apoptosis. To test this, cultures of DLD-1 cells were stained for 72 h with Hoechst 33258, a DNA-sensitive fluorochrome, to assess changes in nuclear morphology following LC3B knockdown. After knockdown of LC3B for 72 h, DLD-1 cells underwent morphologic changes typical of apoptosis, for example, chromatin condensation and a shrunken nucleus (Figure 3(e)).

To determine the form of LC3B knockdown-induced cell death, western blot analysis was performed to assess whether caspase-3 activation was involved in LC3B knockdown. Caspase-3 has a key role in apoptosis, being responsible for the proteolytic cleavage of many key proteins. Processing of caspase-3, measured by the presence of the p17 fragment, was evident after 24 h of treatment with LC3B siRNA. Our results suggest that LC3B knockdown induced apoptosis mediated by caspase-3 activation. 

Next, we hypothesized that PSF interacts with PPAR*γ* and that LC3B is a downstream effector of this interaction in DLD-1 cells. To determine the role of PPAR*γ* in regulating LC3B expression during the proliferation of DLD-1 cells, the expression of PPAR*γ* was knocked down using siRNA. As shown in Figure 4(a), knockdown of PPAR*γ* expression in DLD-1 cells using siRNA was effective, as evidenced by western blot analysis using an anti-PPAR*γ* antibody. To test the functionality of endogenous PPAR*γ*, we transfected DLD-1 cells with a luciferase reporter plasmid. After treatment with rosiglitazone, a PPAR*γ* agonist, we observed increased luciferase activity (Figure 4(b)). We then examined the effect of rosiglitazone (10 *μ*M) on LC3B expression and proliferation in DLD-1 cells (Figure 4(c)). Stimulation with rosiglitazone did not inhibit cell proliferation. Furthermore, addition of the selective and irreversible PPAR*γ* antagonists GW9662 and T0070907 did not inhibit cell proliferation. As expected, PPAR*γ* knockdown decreased the expression of LC3B mRNA in a time-dependent manner. These results suggest that PPAR*γ* activation is not involved in PSF-mediated PPAR*γ* in DLD-1 cells. We provide evidence that PPAR*γ* plays a central role in PSF-dependent regulation of LC3B expression. These data also indicate that a mechanism other than PPAR*γ* activation regulates the PSF-LC3B axis (Figure 4(d)). Taken together, our research may provide a clue to the biological functions of LC3B, and the identified proteins may provide a better understanding of the events involved in colon cancer. It will be of great interest to test this novel anticancer strategy in future *in vivo* studies. The effect of PSF on the functions of LC3B targets and their contribution to PSF-mediated cellular processes requires further investigation.
